# Enhancing Thermo-Mechanical Properties of Epoxy Composites Using Fumed Silica with Different Surface Treatment

**DOI:** 10.3390/polym13162691

**Published:** 2021-08-12

**Authors:** Kyung-Min Kim, Hoon Kim, Hyun-Joong Kim

**Affiliations:** 1 Laboratory of Adhesion & Bio-Composites, Department of Agriculture, Forestry and Bioresources, Seoul National University, Seoul 08826, Korea; min913@snu.ac.kr; 2 Research Institute of Agriculture and Life Sciences, College of Agriculture and Life Sciences, Seoul National University, Seoul 08826, Korea; c12o2cl4@snu.ac.kr

**Keywords:** silica nanoparticle, epoxy/fumed silica composites, thermal stability, thermo-mechanical properties

## Abstract

The objectives of this study are to improve the thermal and mechanical properties of epoxy/fumed silica composite with different surface treatments of fumed silica. The addition of silica nanoparticles improved the thermal stability of the composite and slowed down the pyrolysis process. The crosslinking density and T_g_ of the epoxy/fumed silica composites increased because of the interfacial interaction between the PDMS-treated fumed silica particles and the epoxy matrix. The flexural strength of the epoxy nanocomposite was very high even at a low silica content because of the strong interactions between the PDMS-treated fillers and the epoxy matrix. These strong interfacial interactions originate from the attractive forces between the polymer and the filler. Therefore, the polymer nanocomposite containing the PDMS-treated fumed silica is shown to be sufficiently commercially promising.

## 1. Introduction

In engineering structures, components such as factory piping are exposed to high temperatures; hence, these materials should exhibit high durability, chemical resistance, and thermal stability. As an important requirement for industries, design of high-strength materials with heat resistance has been prioritized [1]. Polymer-based composites are the most widely used materials for structural applications because of their easy processability and low costs. Polymer/inorganic filler composites have received considerable scientific and industrial attention over the past few decades because of their unique properties originating from the combined advantages of inorganic materials and polymers [2,3,4,5]. The dispersion degree of inorganic fillers and the interfacial interaction between the polymer matrix and fillers are important factors to be considered for the preparation of polymer/fumed silica composites. The interfacial cohesive forces of the filler and the polymer matrix have a direct effect on the interfacial stress transfer of the composite structure; therefore, they significantly affect the thermo-mechanical properties of the composite. The high surface-to-volume ratio of the nanomaterial increases the interfacial interaction between the nanofiller and the matrix. Various methods have been used to enhance the interfacial interaction, and numerous studies have been conducted to improve the chemical activity of the filler surface or increase the surface area [6,7,8,9]. Among the available polymer/fumed silica composites, polymer/silica composites have been studied extensively. In addition, there is a method of synthesizing silica filler in one-pot or blending method for manufacturing a polymer/silica composite material [10,11,12]. In order to improve the dispersibility of fillers in polymeric binders, Battistella, M. et al., conducted a study on the chemical surface treatment of silver alumina fillers. Shirono, H. et al., conducted a study on the length of alkyl chains introduced to the surface of silver fumed silica, and El-Fattah, M.A. et al., reported the results of a study on the effect of physical properties on filler size. With reference to these prior studies, a polymer/fumed silica composite was prepared using nano-sized chemically surface-treated fumed silica as a filler, and dispersibility was compared. Because, the interactions between the polymer precursor and the interface can be enhanced through chemical surface treatment of the silica surface. Fumed silica is hydrophilic owing to the presence of silanol group (Si–OH), and it has a low dispersibility owing to particle aggregation; these are major disadvantages [13,14,15]. The hydroxyl groups present on the silica surface can be transformed into organic compounds or polymers through chemical modification. The physical properties, i.e., dispersibility and interfacial interactions, can be improved using fumed silica particles with polydimethylsiloxane (PDMS) surface treatment [16,17].

The binders used in most polymer composites are composed of a thermosetting resin and epoxy. Epoxy resins are widely used in various industrial applications, such as paints, coatings, composite materials, 3D printing, and adhesives, because of their excellent mechanical properties, heat resistance, insulation, and adhesive strength. Moreover, it is possible to control their curing rate by adding various curing agents. However, low conductivities and high coefficients of thermal expansion are the major disadvantages of epoxy resins. Further, this material has a weak external-impact withstanding capability because of its high crosslinking density due to the formation of a 3D network structure. Therefore, when an epoxy composite is used in combination with a material having a different coefficient of thermal expansion, such as a metal, peeling or cracking may occur because of their different thermal deformations. In recent times, many studies have been conducted to improve the fluidity and enhance the impact reinforcement effects of epoxy resins by introducing fumed silica particles [18,19,20,21,22]. These particles serve as inorganic fillers to produce epoxy nanocomposite materials. However, only a few studies on the comparison of thermal and mechanical properties of epoxy nanocomposites have been reported for their application in structures exposed to high temperatures.

In this study, fumed silica with different surface properties was physically dispersed in an epoxy resin, and then the viscosity of the resulting mixture was measured to confirm the dispersibility of fumed silica in epoxy resin. The curing behavior of the mixture was analyzed by differential scanning calorimetry (DSC). The thermal stability of the cured epoxy nanocomposite was confirmed through thermogravimetric analysis (TGA), and its glass transition temperature (T_g_) and crosslinking density were determined by dynamic mechanical analysis (DMA). Moreover, a bending test was performed using a universal testing machine to study the mechanical properties of the epoxy nanocomposite.

## 2. Materials and Methods

### 2.1. Materials

**Figure 1:**
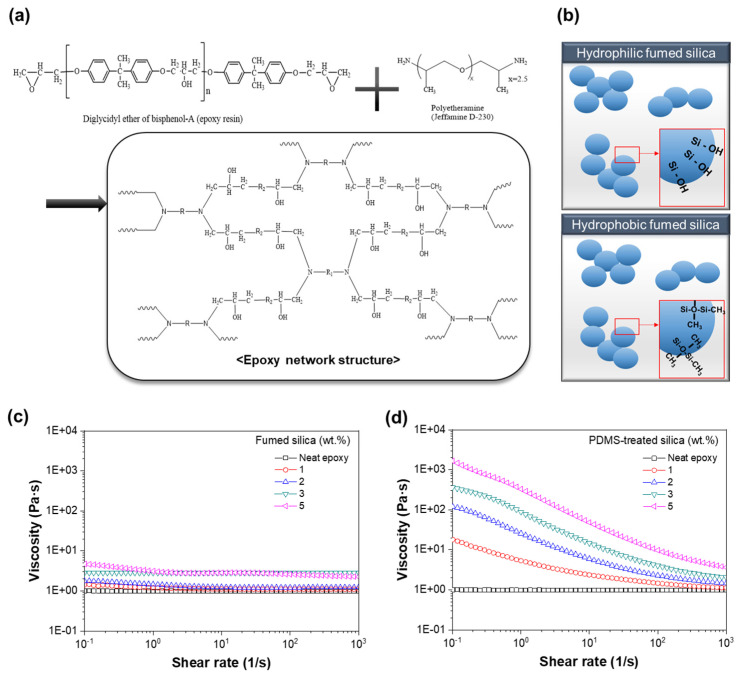
(**a**) Molecular structures of the epoxy resin and polyether diamine crosslinking agent and schematic of the molecular epoxy network structure. (**b**) Schematics of the hydrophilic fumed silica (Aerosil^®^ 150) and hydrophobic fumed silica (Aerosil^®^ R202) structures. Steady shear viscosities of silica/epoxy resin as a function of filler fraction: (**c**) Hydrophilic fumed silica and (**d**) hydrophobic PDMS-treated silica.

A bisphenol A-type epoxy resin (YD-115, E.E.W. 187 g/eq, KUKDO CHEMICAL CO., Seoul, Republic of Korea) containing diglycidyl ether of bisphenol A (DGEBA) with butyl glycidyl ether (BGE) was used. Butyl glycidyl ether (BGE) and polyether diamines (JEFFAMINE^®^ D-230, A.H.E.W. 60 g/eq, HUNTSMAN Corporation, Salt Lake City, TX, USA ) were used as the curing agents. The molecular structures of the epoxy and polyether diamines are shown in Figure 1a.

Fumed silica (AEROSIL^®^150; surface area: 150 ± 15 m^2^/g) and PDMS-treated fumed silica (AEROSIL^®^R202; surface area: 100 ± 20 m^2^/g) were provided by Evonik Industries (Essen, Germany).

### 2.2. Preparation of Epoxy Fumed Silica Composites

The epoxy/fumed silica composite was produced by uniformly dispersing fumed silica in an epoxy resin and then adding a curing agent to the mixture. The equivalent ratio, curing agent:epoxy, was fixed at 1:1.24 (Table 1).

First, fumed silica was dispersed in an appropriate amount of the epoxy resin at 2000 rpm for 10 min by using a paste mixer (ARV-310, Thinky, Tokyo, Japan), followed by stirring at 23 °C for 3 min and ultrasonication (VCX 750, Ultrasonic Processor, Sonic & Materials, Newton, CT, USA). After cooling, a curing agent was added and the defoaming process was performed for 1 min. Next, epoxy/fumed silica mixture with the curing agents was initially cured at 80 °C in a Teflon sheet for 3 h, and the solvent was evaporated, followed by post-curing at 150 °C for 2 h (Figure S1).

### 2.3. Characterization and Measurements

Fourier transform infrared (FTIR) spectroscopy was conducted between 4000 and 400 cm^−1^ using a BERTEX80 FTIR spectrometer (BRUKER, North Billerica, MA, USA) to identify the functional groups present in fumed silica. After the sample was dried at 160 °C for 24 h, a KBr pellet was prepared for the FTIR characterization. The number of scans was 32, and the resolution was 4 cm^−1^. Field-emission scanning electron microscopy (FESEM; SUPRA 55VP, Carl Zeiss, Jena, Germany) was employed to analyze the structure of fumed silica (Figure S2). The morphology of the epoxy/fumed silica composites was also investigated by high-resolution transmission electron microscopy (TEM; JEM-2100F, JEOL Ltd., Tokyo, Japan). The specimens for TEM analysis were cut using an ultramicrotome (EM UC7, Wetzlar, Germany) fitted with a diamond knife, and the corresponding TEM images were obtained at an acceleration voltage of 200 kV.

The difference in the dispersibilities of the fumed silica that underwent different surface treatments was determined by measuring the viscosity of the untreated and the treated fumed silica and epoxy resin mixture at room temperature using a rheometer (MCR-302, Anton Paar, Ostfildem, Germany) equipped with parallel plates, each with a diameter of 25 mm. Steady shear measurement was then performed at a shear rate range of 0.01 to 10^3^ s^−1^, and the results were analyzed to obtain the rheological properties of the fumed silica–epoxy resin mixture.

To examine the thermal stability of the composites, TGA of the epoxy/fumed silica composites was performed using a TGA 4000 system (Perkin Elmer, Waltham, MA, USA). The TGA measurements were conducted at a heating rate of 10 °C/min, over a temperature range of 30–800 °C, under a constant nitrogen flow atmosphere. Specimens were measured only once for each content. The curing behavior of the epoxy/fumed silica and epoxy/PDMS-treated fumed silica was examined by DSC (Q200, TA Instruments, New Castle, DE, USA) at a heating rate of 10 °C/min (from 30 to 300 °C) under a nitrogen flow of 20 mL/min. Three specimens were measured.

The temperature dependences of the dynamic storage modulus (G’) and tan δ values of the epoxy/fumed silica composites were evaluated by DMA (Q800, TA instruments, New Castle, DE, USA) in the film-tension mode over a temperature range of 35–200 °C, at a heating rate of 5 °C/min, strain rate of 0.1%, and frequency of 1 Hz. The dimensions of the specimens were 60 mm × 12 mm × 3 mm. The flexural properties of the composites were determined using a three-point bend test. The epoxy/fumed silica composites were measured using a 10 kN universal testing machine (Z010, Zwick, Ulm, Germany) at a crosshead speed of 0.1 mm/s. The flexural specimens had dimensions of 60 mm× 12 mm × 3 mm. The tests were performed at a temperature of 25 ± 1 °C and humidity of 50 ± 2% RH, in accordance with the ASTM D790 standard methods.

## 3. Results and Discussion

### 3.1. Characterization

In this study, a DGEBA-type epoxy resin diluted in BGE was used as the epoxy resin, and polyether diamines were used as the crosslinkers. Figure 1a shows the schematic of the resulting epoxy network structure [23]. Fillers with different surface functional groups were selected for the experiment (Figure S2) [24].

As shown in Figure 1b, the fumed silica without surface treatment contains a silanol group (Si–OH) as the functional group, whereas the PDMS-treated fumed silica has exposed methyl groups, as confirmed via FTIR analysis (Figure S3). The FTIR spectrum of the untreated fumed silica showed characteristic asymmetric stretching, symmetric stretching, and bending vibrations of the Si–O–Si band at 1103, 804, and 472 cm^−1^, respectively. The spectrum also showed absorption peaks at 3435 and 1631 cm^−1^, which could be assigned to the stretching and bending vibrations of the OH group on the silica surface. In the FTIR spectrum of the PDMS-treated fumed silica, additional peaks at 2960 (assigned to C–H stretching in CH_3_), 1262 (CH_3_ symmetric bending in Si–CH_3_), and 804 cm^−1^ (-CH_3_ rocking in Si–CH_3_) were observed, which confirmed the existence of different functional groups in the PDMS-treated fumed silica [15,23,25,26,27].

After sufficiently dispersing the fumed silica with different surface functional groups in the epoxy resin (YD-115) through mixing and ultrasonication, the steady shear viscosities of the mixtures with different silica contents were measured (Figure 1c,d). As shown in Figure 1c, the untreated silica suspensions exhibited Newtonian fluid behavior, whereas the viscosities of the epoxy/PDMS-treated silica mixtures showed a strong dependence on the silica content, as shown in Figure 1d. The viscosity of the mixtures decreased with increase in the shear rate. This characteristic, called the shear thinning behavior, is typical of non-Newtonian fluids. Shirono H. et al., measured the viscosity of fumed silica by surface treatment with silanes with different alkyl chain lengths on the surface. Suspensions of untreated fumed silica and low chain length treated fumed silica showed Newtonian behavior. However, strong shear thinning behavior was observed in the surface-modified fumed silica with longer alkyl groups. Additionally, Mai, V.D. et al., showed a shear thinning behavior in which the viscosity of the epoxy resin/nano-Al_2_O_3_ composite material decreased as the shear rate increased [14,28]. Compared with the results of published literature, it shows similar research results in which the behavior of Newtonian and non-Newtonian fluids according to the different surface treatment of the filler. The shear thinning behavior of the mixtures can be attributed to the redistribution of fillers and their directions.

### 3.2. Curing Behavior

**Figure 2:**
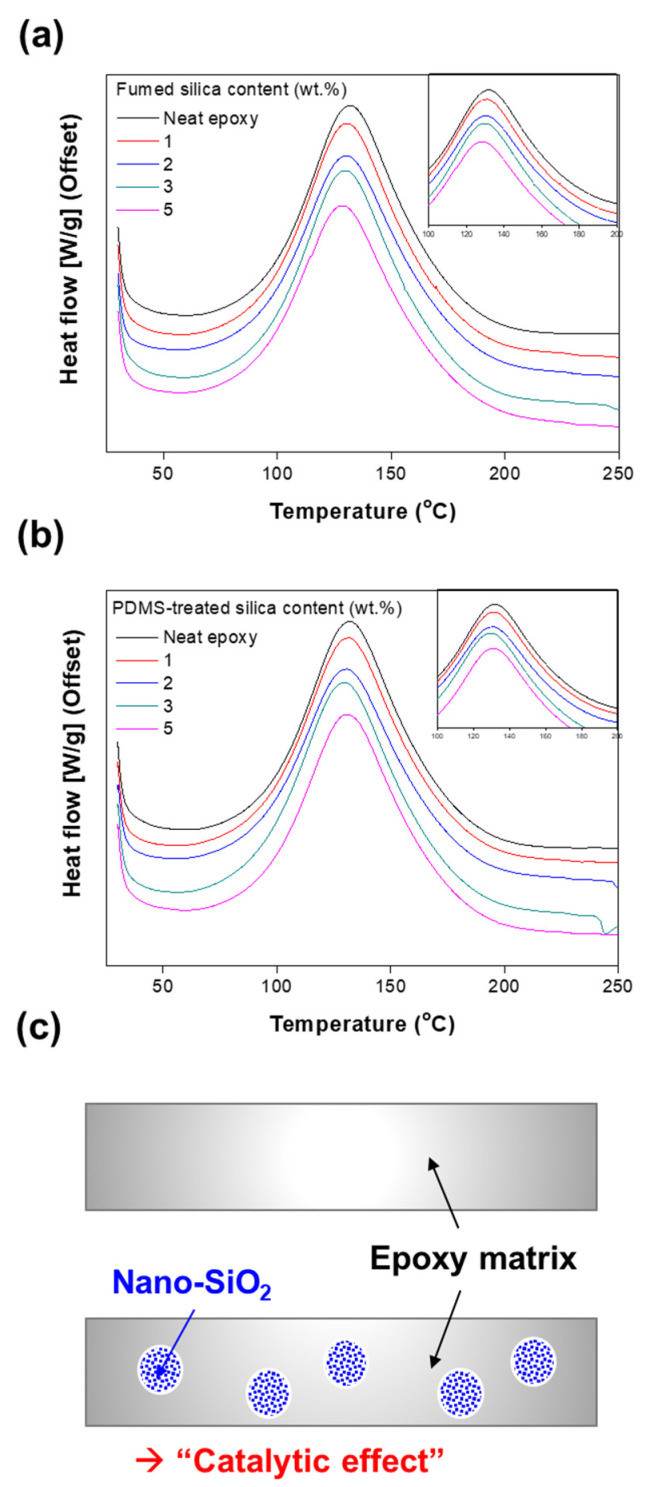
DSC curves of epoxy nanocomposites with (**a**) fumed silica and (**b**) PDMS-treated fumed silica. (**c**) Schematic of the catalytic effect.

The curing reaction of the epoxy composites was investigated by DSC. Figure 2a,b show the DSC thermograms of the cured epoxy and its composites for the untreated and the different PDMS-treated fumed silica contents. Evidently, the peak maximum temperature, defined as T_p_, decreased with increase in the epoxy/fumed silica composite filler content. The T_p_ decreased from 132.5 to 129 °C as the PDMS-treated fumed silica content was increased from 1 to 5 wt.%, respectively, as shown in Figure S4. The peak maximum is shifted toward the lowest temperatures for the composites with 5 wt.% of untreated fumed silica and PDMS-treated fumed silica. This indicates that fumed silica has a catalytic effect on the curing reaction of the epoxy composite (Figure 2c). Jiang W. et al., Jin, F.L. et al., and Omrani, A. et al. reported similar observations of catalytic effect using the epoxy/alumina nanoparticle composites [27,29,30]. This means that high weight percent of nanoparticle filler has a converse effect on the polymer network formation. It could be described by topological restrictions produced during the epoxy network evolution. This only refers to the behavior that has an adverse effect on network formation during the curing process of the epoxy/nanocomposite. These results also confirm that the polymer chains are constrained by the interface-particle interactions [24,31,32,33].

### 3.3. Morphology

**Figure 3:**
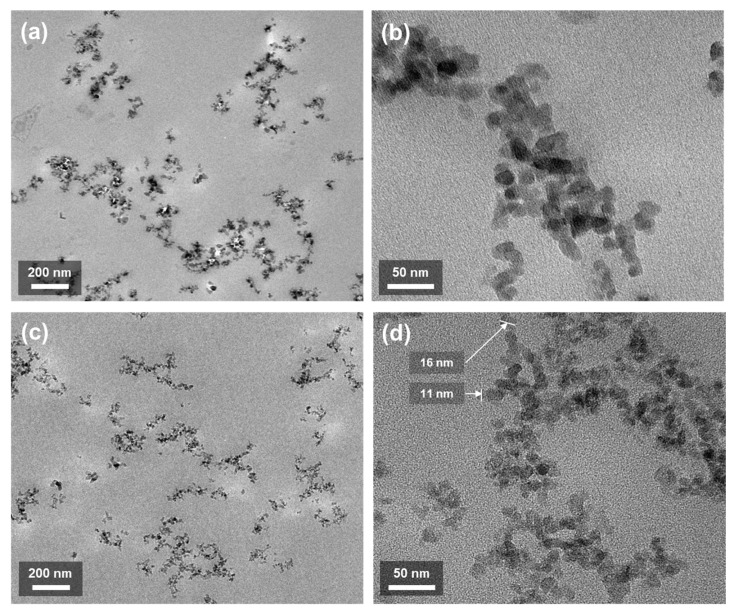
TEM images of the epoxy/fumed silica composite with 3 wt.% (**a**,**b**) fumed silica and (**c**,**d**) PDMS-treated silica.

The morphological characteristics of the epoxy/fumed silica composites with 3 wt.% fumed silica were analyzed by TEM. Figure 3a,b show the TEM images of the untreated fumed silica, and Figure 3c,d show those of the surface-treated fumed silica. Evidently, the specific surface areas of the untreated and the treated fumed silica were similar. As shown Figure 3a,c, both fumed silica and PDMS-treated silica particles are uniformly dispersed. However, the sizes of the untreated fumed silica particles observed in Figure 3b are larger than those of the treated fumed silica particles in Figure 3d. This is because the silanol (Si–OH) group on the surface of the untreated fumed silica particles allows moisture absorption [6,31,32,33].

### 3.4. Thermal Stability

**Figure 4:**
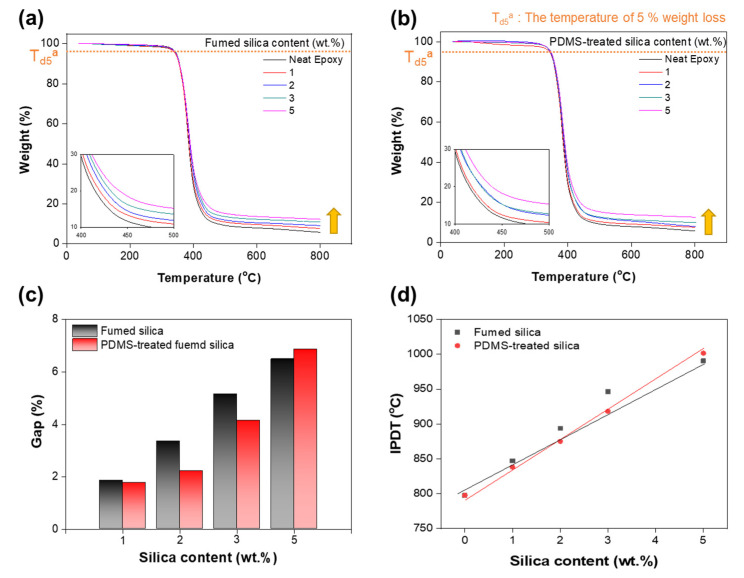
Thermograms of epoxy composites with (**a**) fumed silica and (**b**) PDMS-treated fumed silica. (**c**) Gap of char weight percent at 800 °C. (**d**) Integral procedural decomposition temperature (IPDT) of the epoxy/fumed silica composites.

To determine the thermal stability, TGA of the epoxy/fumed silica composite was performed. The TGA curves of the pure epoxy and epoxy/fumed silica composites showed similar thermal degradation tendencies as evident from Figure 4a,b. In general, the maximum mass loss occurred at approximately 300–600 °C. The addition of silica nanoparticles improved the thermal stability of the composite and delayed the thermal decomposition process. At 800 °C, the amount of char increases with increase in the fumed silica content. However, the char yield is not 0% for the pure epoxy at 800 °C. This may be attributed to the carbonization of the epoxy matrix and the presence of unreacted substances [34,35]. The difference between the char yields of the pure epoxy and the epoxy/fumed silica composites, produced at 800 °C, was defined as “Gap” to assess the rate of formation of char with increase in the filler content.

Gap = W_f_ − W_0_ (1)

where W_f_ is the char yield (%) of epoxy/fumed silica composites, and W_0_ is the char yield (%) of pure epoxy. The untreated fumed silica rapidly formed char upon heating to high temperatures. Owing to the good dispersibility of PDMS-treated fumed silica, only a small amount of char is formed in the beginning; however, there is a steep increase in the char formation when the silica content is increased to 5 wt.%.

Td_5_ was defined as the temperature at which the mass percentage of the epoxy/fumed silica composite decreased by approximately 5% (Figure S5a,b). Figure S5d shows the TGA results of the pure fumed silica filler. The mass of the untreated fumed silica was reduced by approximately 2%. The mass of the epoxy/surface-treated fumed silica composite decreased rapidly above 300 °C, and a char yield of approximately 88% was obtained at 800 °C. In the case of the epoxy composite with PDMS-treated fumed silica, the Td_5_ should be actually low owing to the decomposition of organic matter on the surface. However, there were no significant differences in the Td_5_ values of the epoxy/surface-treated fumed silica composites. This suggests that the PDMS-treated fumed silica undergoes uniform dispersion in the epoxy resin to form a complex.

The integral procedural decomposition temperature (IPDT), proposed by Doyle, is related to the volatile fraction of polymers and can be utilized to estimate the overall intrinsic thermal stability of polymers during decomposition. In this study, the IPDT was calculated from the TGA curve using the following equations [36,37,38,39]:

IPDT = A*K*(T_f_ − T_i_) + T_i_, (2)

A* = (S1 + S2)/(S1 + S2 + S3),(3)

K* = (S1 + S2)/S1,(4)

where A* is the area ratio of the total experimental curve divided by the total TGA thermogram, K* is the modulus, T_i_ is the initial experimental temperature (35 °C), and T_f_ is the final experimental temperature (800 °C). For the analysis, the TGA plot was divided into three regions represented by S1, S2 and S3, as shown in Figure S5c. The linear line was drawn from point IPDT at 0 wt.% to point IPDT at 5 wt.% in Figure 4d.

Jin, F.L. et al. and Chiang, C.L. et al. showed that nano inorganic particle improve the thermal stability of pure epoxy. Liu, Y.L. et al. reported epoxy-silica hybrid composites have an increase in IPDT with increasing silica content. It means that silica particles contribute to the level-up of IPDT while delaying the thermal decomposition rate of the organic part of the organic-inorganic hybrid composite [26,36,39]. The IPDTs of untreated and treated fumed silica-epoxy composites increased with increase in the silica content. At low filler contents, the difference in the IPDTs of the untreated and treated silica/epoxy composites was negligible. At a high silica content (5 wt.%), the IPDT of the PDMS-treated silica/epoxy composite was approximately 10 °C higher than that of the untreated silica/epoxy composite. Compared with the published literature, the thermal stability of the silica weight fraction of the epoxy/thin silica nanocomposite has similar observations. In contrast to the influence of the silica weight fraction, the difference value of IPDT on the surface treatment of the filler is low. However, It indicated that the PDMS-treated silica/epoxy composite shows better thermal stability (Figure S5a,b).

### 3.5. Thermo-Mechanical Properties

**Figure 5:**
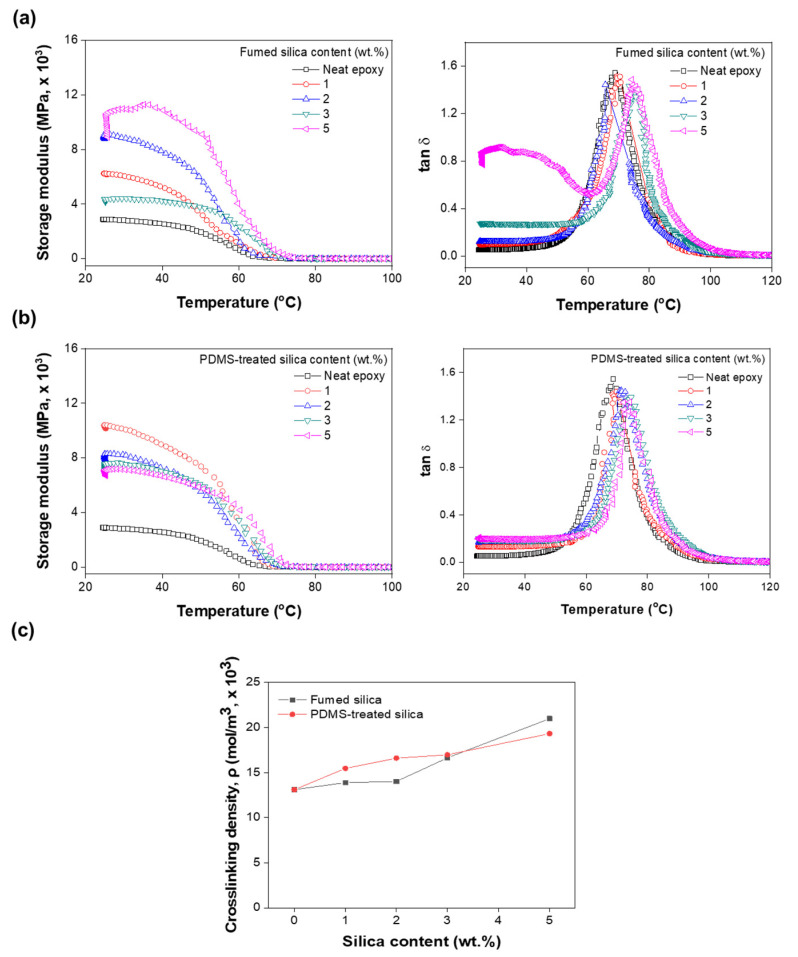
Variations in the storage modulus and tan 𝛿 values of the epoxy composites as functions of (**a**) fumed silica and (**b**) PDMS-treated fumed silica contents. (**c**) Crosslinking densities of epoxy/fumed silica and epoxy/PDMS-treated silica composites with silica content.

The effect of silica nanoparticles with different surface functional groups on the viscoelastic properties of the crosslinked polymers was analyzed using DMA (Figure 5). The storage modulus and tan 𝛿 of the pure epoxy and composites showed temperature dependence (Figure 5a,b). Figure 5a,b show that the T_g_ of the epoxy composite containing silica nanoparticles is higher than that of the pure epoxy resin by approximately 5 °C [40,41,42,43,44]. Figure 5a shows that the storage modulus at the initial temperature increased as the untreated fumed silica filler content increased. However, the storage modulus at the initial temperature of the PDMS-treated fumed silica/epoxy composite was not dependent on the filler content. The results for epoxy/fumed silica composite confirmed that the storage modulus in the rubbery region increased as the filler content increased (Figure S6a,b). The crosslinking density of the epoxy/fumed silica composites with different surface functional groups was calculated, and the results are shown in Figure 5c. Evidently, the crosslinking density of the PDMS-treated silica composite is slightly higher than that of the untreated silica composite. The crosslinking density and T_g_ of the epoxy/fumed silica composites increased because of the PDMS treatment-induced interfacial interactions between the fumed silica particles and the epoxy matrix.

**Figure 6:**
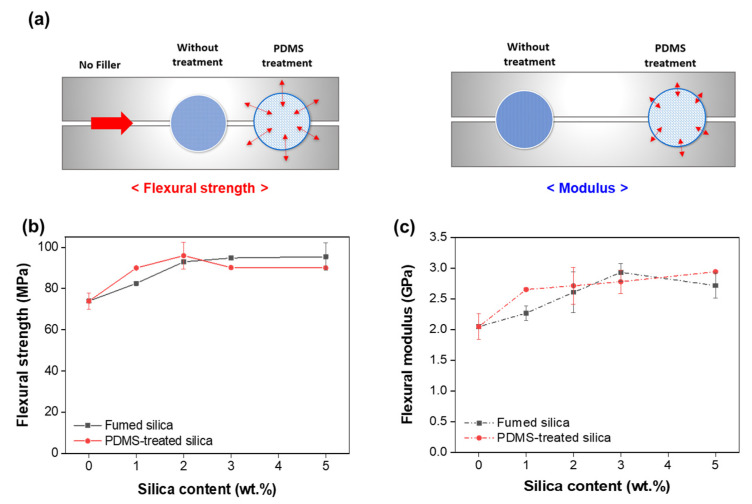
(**a**) Illustration of mechanical properties. Variations in the (**b**) flexural strength and (**c**) flexural modulus of the epoxy/fumed silica composites as functions of silica content.

Flexural tests were performed to confirm the mechanical properties of the epoxy composite material. The interaction between the nanofiller particles and the matrix was confirmed by applying a continuous force to the structure. Figure 6b,c show that the flexural strength and modulus increase with increase in the filler content for untreated fumed silica composite. In the PDMS-treated fumed silica/epoxy composite, the flexural strength is maximum at 2 wt.% and then decreases; however, the modulus continues to increase. The attraction between the polymer and the filler gave rise to the observed flexural strength of the composite, leading to an increase in its maximum bending strength. In particular, the PDMS-treated composite showed maximum strength at a low silica content owing to the strong interaction of the fumed silica particles with the polymer matrix. The flexural modulus of a material is defined as the stress that resists deformation in the material. The modulus of elasticity of the PDMS surface-treated fumed silica/epoxy composite is slightly higher than that of the untreated fumed silica/epoxy composite. The obtained flexural modulus indicates weak interaction between the filler and the polymer matrix, contrary to the case of flexural strength (Figure 6a).

## 4. Conclusions

The dispersibility of fumed silica particles in a polymer matrix was confirmed by measuring the viscosity of a mixture of fumed silica and epoxy resin with different surface properties. Depending on the surface treatment of fumed silica, shear thinning behavior and Newtonian behavior was observed in the composite. In particular, the initial viscosity of the PDMS-treated silica mixture increased with increase in the silica content. In the curing reaction of the mixture, the maximum temperature at the exothermic peak decreased as the content of the fumed silica filler was increased. These results show that the fumed silica filler has a catalytic effect on the epoxy curing reaction. Additionally, the Td_5_, char content, and IPDT of the composites were analyzed. The addition of silica nanoparticles improved the thermal stability of the composite and slowed down the pyrolysis process. The crosslinking density and T_g_ of the epoxy/fumed silica composites increased because of the interfacial interaction between the PDMS-treated fumed silica particles and the epoxy matrix. The flexural strength of the epoxy nanocomposite was very high even at a low silica content, because of the strong interactions between the PDMS-treated fillers and the epoxy matrix. These strong interfacial interactions originate from the attractive forces between the polymer and the filler. Therefore, surface-treated fumed silica is a promising filler material for industrial applications.

## Figures and Tables

**Table 1:** Formulation of epoxy/fumed silica composites.

Sample	Epoxy Resin (g)	Curing Agent (g)	Filler	
			(g)	(wt.%)
Pure epoxy	10		0	0
Ep/F1			0.14	1
Ep/F2		4	0.29	2
Ep/F3			0.43	3
Ep/F5			0.74	5

Curing agent:epoxy resin ratio = 1:1.24. Filler used—Silica nanoparticle (AEROSIL^®^150, AEROSIL^®^R202).

## Data Availability

Data is contained within the article.

## Supplementary Materials

The following are available online at https://www.mdpi.com/article/10.3390/polym13162691/s1, Figure S1: Specimens images of (**a**) Epoxy/fumed silica composites (**b**) Epoxy/PDMS-treated composites, Figure S2: FESEM images of (**a**) hydrophilic fumed silica and (**b**) hydrophobic PDMS-treated fumed silica nanoparticles, Figure S3: FTIR spectra of fumed silica and PDMS-treated fumed silica nanoparticles, Figure S4: DSC results of (**a**) epoxy/fumed silica composites and (**b**) epoxy/PDMS-treated fumed silica composites, Figure S5: TGA results of (**a**) Epoxy/fumed silica composites (**b**) Epoxy/PDMS-treated composites. (**c**) Schematic representation of S1, S2, and S3 for A* and K*. (**d**) TGA curves of fumed silica nanoparticles, Figure S6: (**a**) DMA results of the epoxy/fumed silica composites. (**b**) DMA results of the epoxy/PDMS-treated fumed silica composites. (**c**) Storage modulus of the epoxy/fumed silica composites. (**d**) Storage modulus of epoxy/PDMS-treated fumed silica composites.
