# Supplementary material for: Enhancing Thermo-Mechanical Properties of Epoxy Composites Using Fumed Silica with Different Surface Treatment
Source: Polymers (Basel). 2021 Aug 12;13(16):2691. doi: 10.3390/polym13162691 (PMC8400810; doi:10.3390/polym13162691)
Supplement: Supplementary file 1 [file polymers-13-02691-s001.zip › polymers-1311822-supplementary.pdf]

Supporting Information

Enhancing Thermo-Mechanical Properties of Epoxy Composites using Fumed silica with Different Surface Treatments

*Kyung-Min Kim[a], Hoon Kim[b], Hyun-Joong Kim[a,b]**

[a] *Lab. Of Adhesion & Bio-Composites, Department of Agriculture, Forestry and Bioresources,*

[b] *Research Institute of Agriculture and Life Sciences, College of Agriculture and Life Sciences,*

Seoul National University, Seoul 08826l, Republic of Korea.

*Corresponding author, Fax: +82 28732318, E-mail address: hjokim@snu.ac.kr (H.-J. Kim)

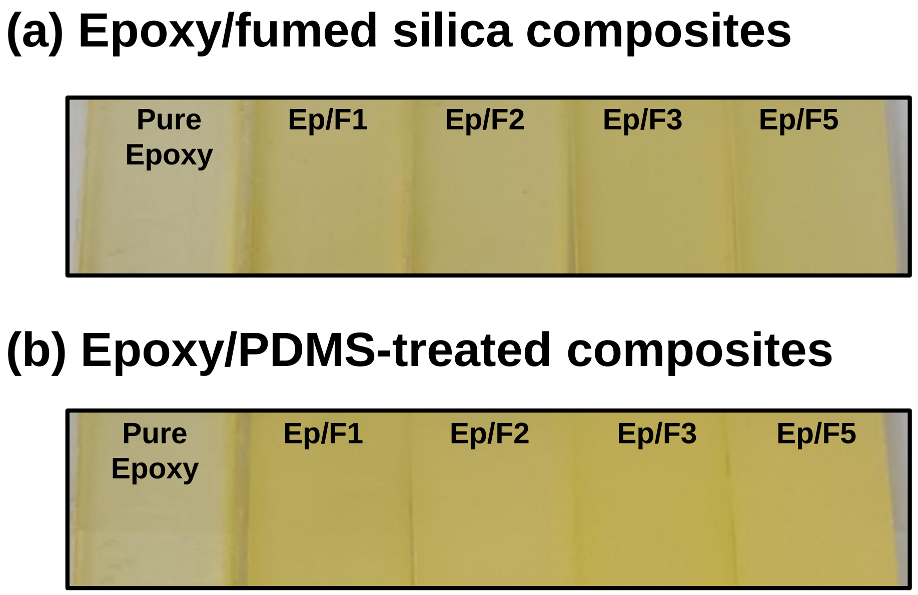

Figure S1. Specimens images of (**a**) Epoxy/fumed silica composites (**b**) Epoxy/PDMS-treated composites**.**

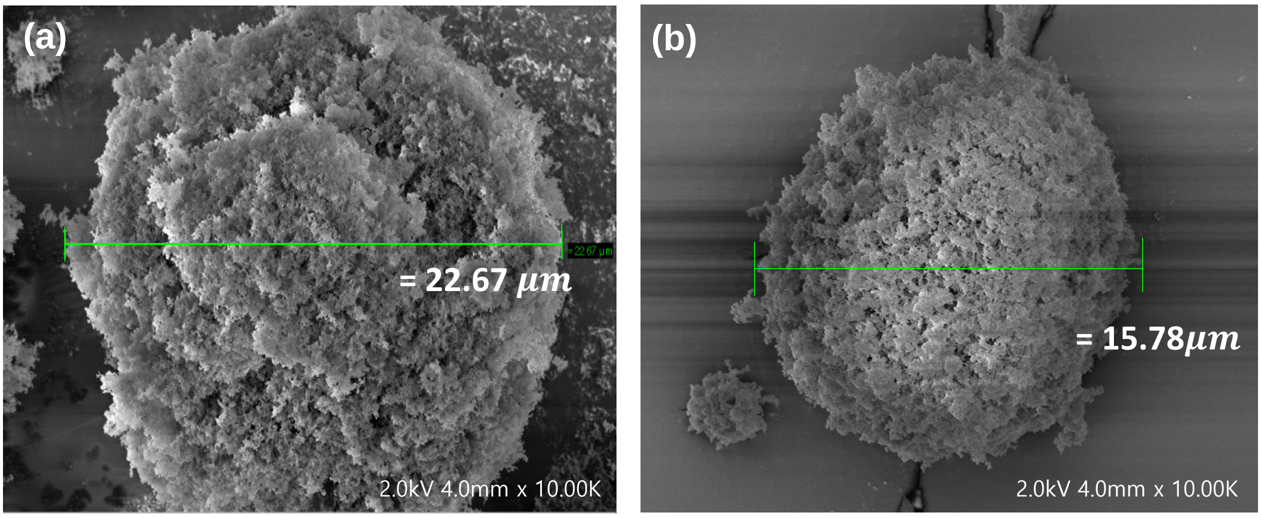

Figure S2. FESEM images of (**a**) hydrophilic fumed silica and (**b**) hydrophobic PDMS-treated fumed silica nanoparticles.

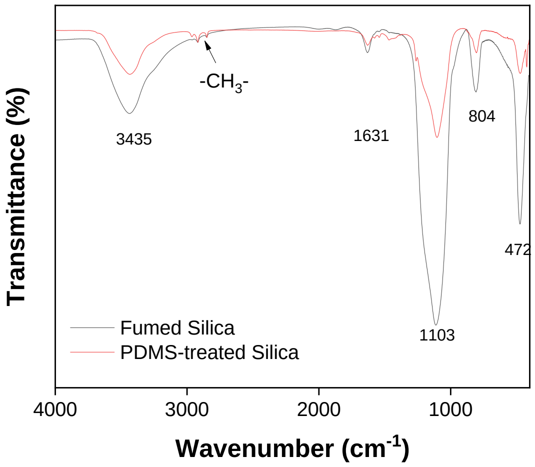

Figure S3. FTIR spectra of fumed silica and PDMS-treated fumed silica nanoparticles

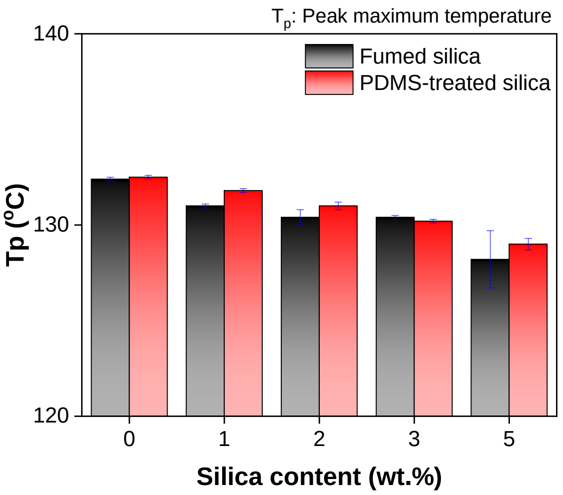

Figure S4. DSC results of (**a**) epoxy/fumed silica composites and (**b**) epoxy/PDMS-treated fumed silica composites.

(a)

Fumed silica contents (wt.%)	T_{d5}[a] (℃)	IPDT[b] (℃)	Char(%) at 800 ℃
0	347	797.6	5.8
1	343.9	847.3	7.6
2	346.9	893.8	9.1
3	346	946.6	10.9
5	345	990.5	12.3

[a]:The temperature of 5% weight loss

[b]:Integral Procedural Decomposition Temperature

(b)

Heating rate: 10 ℃/min

PDMS - treated silica contents (wt.%)	T_{d5}[a] (℃)	IPDT[b] (℃)	Char(%) at 800 ℃
0	347.0	797.6	5.8
1	341.7	838.1	7.5
2	344.4	875.0	8.0
3	346.2	918.1	9.9
5	346.8	1001.6	12.6

(c)

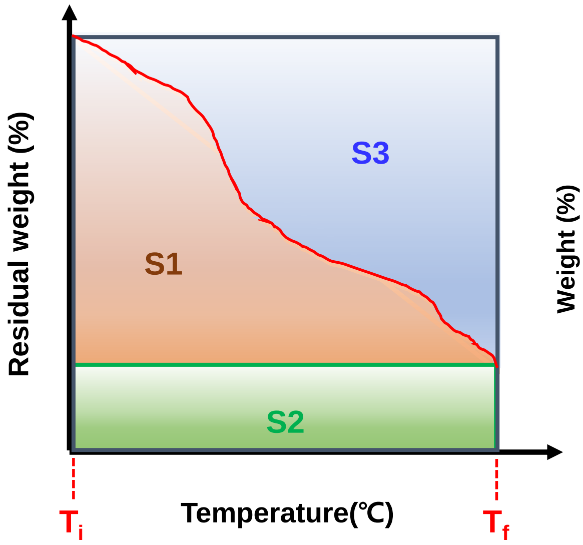

(d)

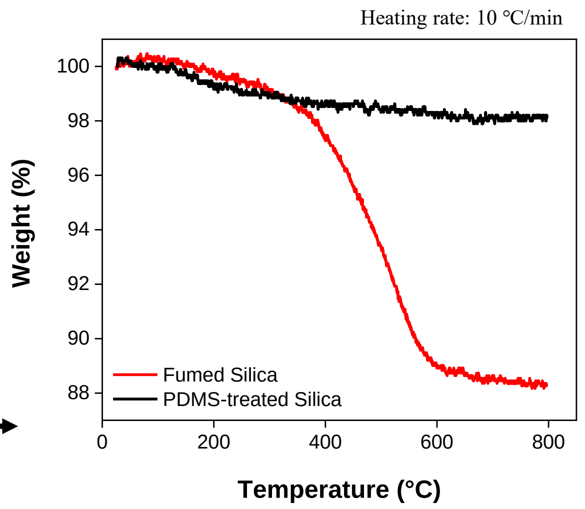

Figure S5. TGA results of (**a**) Epoxy/fumed silica composites (**b**) Epoxy/PDMS-treated composites. (**c**) Schematic representation of S1, S2, and S3 for A* and K*. (**d**) TGA curves of fumed silica nanoparticles.

(a)

Fumed silica content (wt.%)	T_g (°C)	Storage modulus (MPa)		Crosslinking density, ρ [mol/m³]
		Glassy region[a]	Rubbery region[b]	
0	≅ 68	2699	6.9	1309
1	≅ 69	5707	7.2	1388
2	≅ 66	8452	7.7	1402
3	≅ 73	4342	8.2	1662
5	≅ 74	18957	10.4	2083

[a]: Storage modulus at 35 ℃
[b]: Storage modulus at T_g+30 ℃

(b)

Heating rate: 5 ℃/min

PDMS - treated fumed silica content (wt.%)	T_g (°C)	Storage modulus (MPa)		Crosslinking density, ρ [mol/m³]
		Glassy region[a]	Rubbery region[b]	
0	≅ 68	2699	6.9	1309
1	≅ 69	9540	8.0	1544
2	≅ 71	7778	8.3	1659
3	≅ 73	7369	8.3	1697
5	≅ 74	6957	9.4	1932

Figure S6. (a) DMA results of the epoxy/fumed silica composites**. (b**) DMA results of the epoxy/PDMS-treated fumed silica composites.
